# Age-related pattern and monocyte-acquired haemozoin associated production of erythropoietin in children with severe malarial anaemia in Ghana

**DOI:** 10.1186/1756-0500-7-551

**Published:** 2014-08-20

**Authors:** James Abugri, John Kweku Amissah Tetteh, Lateef Adebayo Oseni, Henrietta Esi Mensah-Brown, Rupert Kantunye Delimini, David Osei Obuobi, Bartholomew Dicky Akanmori

**Affiliations:** Infectious Diseases Research Laboratory (IDRL): Department of Biochemistry, Cell and Molecular Biology, University of Ghana, Accra, Ghana; Department of Applied Chemistry and Biochemistry, University for Development Studies, Tamale, Ghana; Immunology Department, Noguchi Memorial Institute for Medical Research, College of Health Sciences, University of Ghana, Accra, Ghana

## Abstract

**Background:**

Malaria continues to be a global health challenge, affecting more than half the world’s population and causing approximately 660,000 deaths annually. The majority of malaria cases are caused by *Plasmodium falciparum* and occur in sub-Saharan Africa. One of the major complications asscociated with malaria is severe anaemia, caused by a cycle of haemoglobin digestion by the parasite. Anaemia due to falciparum malaria in children has multifactorial pathogenesis, which includes suppression of bone marrow activity. Recent studies have shown that haemozoin, which is a by-product of parasite haemoglobin digestion, may play an important role in suppression of haemoglobin production, leading to anaemia. In this study we correlated the levels of erythropoietin (EPO), as an indicator of stimulation of haemoglobin production, to the levels of monocyte acquired haemozoin in children with both severe and uncomplicated malaria. There was a significantly negative correlation between levels of haemozoin-containing monocytes and EPO, which may suggest that haemozoin suppresses erythropoiesis in severe malaria. A multiple linear regression analysis and simple bar was used to investigate associations between various haematological parameters.

**Methods:**

To examine the levels of erythropoietin in the age categories, the levels of erythropoietin was measured using a commercial Enyme-Linked Immunosorbent Assay (ELISA). Giemsa-stained blood smears were used to determine percentage pigment containing monocytes. The haemozoin containing monocytes was expressed as a percentage of the total number of monocytes. To obtain the number of haemozoin containing monocytes/μL the percentage of haemozoin containing monocytes was multiplied by the absolute number of monocytes/μL from the automated haematology analyzer.

**Results:**

The levels of erythropoietin in younger children (<3 years) was significantly higher than in older children with a similar degree of malaria anaemia (Hb levels) (p < 0.005). Haemozoin-containing monocytes were relatively higher in severe malaria anaemia patients compared to those with uncomplicated malaria (p < 0.001).

**Conclusions:**

Age purportedly has a direct effect on background levels of erythropoietin. With corresponding decreased levels of erythropoietin in older children with the same degree of severe malarial anaemia, conceivably, the bone marrows of younger children with acute malaria may be less sensitive to erythropoietin.

## Background

Malaria is an infectious disease and a major public health problem. Malaria can be uncomplicated or severe, often manifesting as severe anaemia, impaired consciousness or respiratory distress [1]. Metabolic complications in severe malaria in children include hypoglycaemia and metabolic acidosis. Major clinical complications of severe malaria in non-immune adults include cerebral malaria, acute renal failure, pulmonary oedema and acute respiratory distress syndrome.

Malaria contributes significantly to the development of anaemia in sub-Saharan Africa and elsewhere, where the disease is prevalent [2]. Indeed severe malarial anaemia (SMA), defined as as anaemia with haemoglobin (Hb) concentration of <5 g/dL with *P. falciparum* asexual parasitemia, in the absence of any other or additional identifiable cause of anaemia [3], is the most widespread complication of *Plasmodium falciparum* infection, especially among children (WHO, 2000). The anaemia of malaria has profound pathological effects on patients and its root cause is multifactorial and encompasses haemolysis, erythrophagocytosis, dyserythropoiesis, and ineffective erythropoiesis [4–6]. Its manifestations are also complex and defy accurate delineation. Blood transfusion as a treatment for malaria has very high risks because of the high incidence of HIV and other blood-borne infections.

In addition to direct destruction of infected erythrocytes by the malaria parasites during schizogony and the premature removal of non-parasitized erythrocytes, reversible suppression of erythropoiesis has also been reported to play a key role in its pathogenesis [7]. Suppression of erythropoiesis means a failure of the bone marrow to respond adequately to the excessive removal of the erythrocytes by producing more erythrocytes and to alleviate the anaemia. The suppression of bone marrow function in severe anaemia patients with microscopically undetectable parasitaemia has been presumptively attributed to subpatent malaria parasites [8]. However, subpatent parasitaemia cannot account fully for the suppression of bone marrow function. Haemozoin and inflammatory mediators have also been implicated in the suppression of bone marrow function [9, 10]. Their roles and the interplay between the various factors in the development of severe malaria anaemia have not been explicitly elucidated. The thrust of this study was therefore to investigate the level of erythropoietin and haemozoin in both severe malaria anaemia and uncomplicated malaria in Ghanaian children. To achieve this, levels of erythropoietin and haemozoin containing monocytes were measured using approved protocols.

## Methods

### Study subjects

This study was hospital-based,, involving children aged 1–13 years, admitted to the emergency unit of the Department of Child Health (DCH) of the Korle-Bu Teaching Hospital and outpatients at the Korle-Bu Polyclinic, both in Accra, Ghana during the peak malaria transmission season (July to August, 2005). The patients were examined clinically by a physician and laboratory investigations including haematology, microscopy and clinical chemistry carried out.

Parents/guardians of patients gave informed consent prior to recruitment. Demographical, clinical history and management/outcomes were also documented. This study formed part of a larger one, which was approved by the Institutional Review Board (IRB) and the Scientific and Technical Committee (STC) of the Noguchi Memorial Institute for Medical Research (NMIMR), College of Health Sciences, University of Ghana, Legon. The study also received the approval of the Scientific and Ethical Review Committee of the University of Ghana Medical School, College of Health Sciences, and University of Ghana.

### Inclusion criteria for malaria patients

Inclusion criteria for malaria patients were fever (≥37.5°C) measured within 24 hours of admission, malaria parasitaemia, and at least one other sign of malaria (vomiting, diarrhoea, malaise etc.). Children with other clinical presentations other than malaria or that had a positive sickling test were excluded from the study. Patients were categorized as uncomplicated and severe malaria based on previously established clinical characterization, haematological and other indices [3, 11].

Severe malaria cases had haemoglobin (Hb) ≤5g/dL and any level of peripheral blood *Plasmodium falciparum* parasitaemia, were fully conscious without severe bleeding or convulsions and had no other cause of anaemia. SMA also included patients who were unconscious with a Blantyre coma score of ≤3, duration of coma >60 minutes, any Hb value, any parasitaemia and no other neurological diseases [3]. Uncomplicated malaria cases were patients who were fully conscious, had malaria parasitaemia and no severe complication as described above. The physician examined the patients clinically and other causes of anaemia such as helminth infection, nutritional deficiency and HIV infection were precluded.

### Blood collection for laboratory analyses

Venous blood was collected into sterile vacutainer tubes containing heparin and EDTA for routine diagnostic purposes. Plasma was obtained by centrifugation using the poor-platelet plasma (PPP) method. In this method, the vacutainer tube containing blood samples was centrifuged at 65 × g for 15 minutes at room temperature. The supernatant which is the Rich-Platelet-Protein (RPP) fraction was taken without disturbing the buffy coat and put into another tube. This was again centrifuged at 960 × g for 10 minutes. The supernatant which is the PPP was then collected and stored at −80 degrees Celsius.

Thick and thin blood films obtained at enrollment prior to therapy were stained with Giemsa for detection, identification and enumeration of Plasmodium. Peripheral parasite density was determined from thick films based on the number of asexual forms against 300 white blood cells (WBCs) and the value multiplied by each individual’s WBC count from the automatic haematology analyzer (SYSMEX, Japan) to obtain parasites per μL of blood. Absolute parasite counts were calculated for each child using either thin films (counting parasitized RBCs per 500 RBCs), or from thick films (counting parasitized RBCs per 300 WBCs).The Quantikine IVD Epo ELISA kit was used to determine erythropoietin levels following the manufacturer’s instructions.

### Statistical analyses

Statistical analyses were carried out using Sigmaplot 12 (http://www.sigmaplot.com) and graphpad prism 5. Spearman’s rank coefficients were calculated to assess correlations between various parameters and levels of haemoglobin as well as levels of haemozoin containing monocytes and neutrophils.

## Results

### Clinical characteristics of study participants

A total of 100 severe and uncomplicated cases of malaria as well as 20 age-matched healthy uninfected individuals, both male and female were involved in this study. They included 36 children with severe malaria and 64 children with uncomplicated malaria. The mean Hb for children with complicated malaria was 3.9 g/dl and the complicated malaria cases overall had a higher mean parasitaemia compared to the uncomplicated malaria cases and the 20 healthy matched controls as shown in (Table 1). Generally, parasitaemia was high in both complicated and uncomplicated malaria cases. However, the difference between the levels of parasitaemia in both groups was not statistically significant (P = 0.722). Relatively, children with complicated malaria were younger than children presenting with uncomplicated malaria (Table 1). The difference in age between the two groups was statistically significant (P <0.001).

**Table 1:** **Clinical characteristics and laboratory parameters of Ghanaian children with complicated and uncomplicated malaria**

Group	N	Mean age (Years)	Mean haemoglobin (g/dL)	Mean parasitaemia (parasites/uL)
**Complicated malaria**	36	4 (1–10)*	3.9 (2.1-4.8)*	175,026.4 (2,832-1,958,270)*
**Uncomplicated malaria**	64	6 (1–13)*	9.8 (8.1-12.7)*	121,692.4 (3,618-516,870.)*
**Controls**	20	6 (2–8)*	11.335 (9.6-12.5)*	302.6 (0.0-2,472)*

*Data are means (minimum and maximum values). N = sample size.

### Erythropoietin and other haematogical correlates

**Figure 1:**
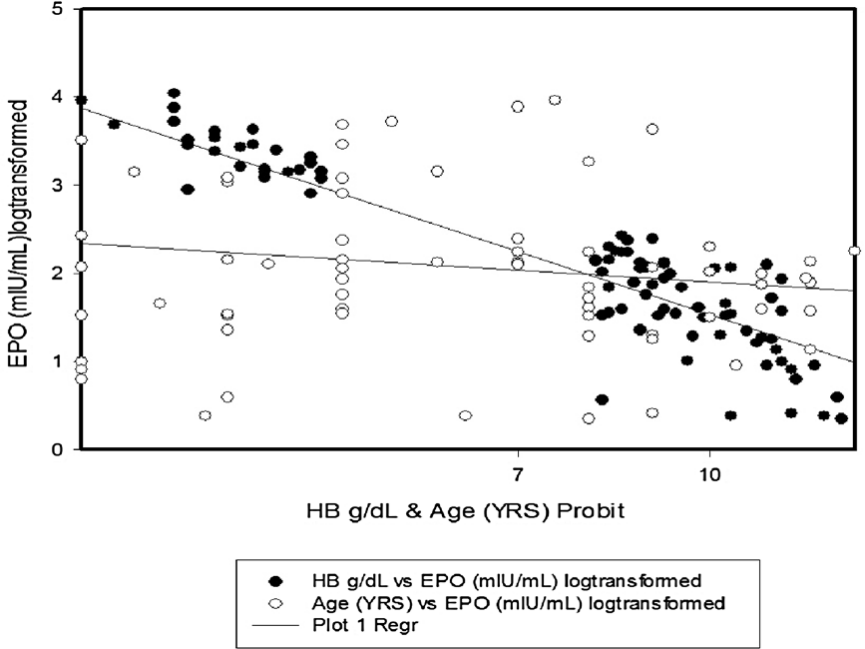
**The relationship between plasma erythropoietin** **(log transformed), haemoglobin levels and age in paediatric malaria cases.**

**Figure 2:**
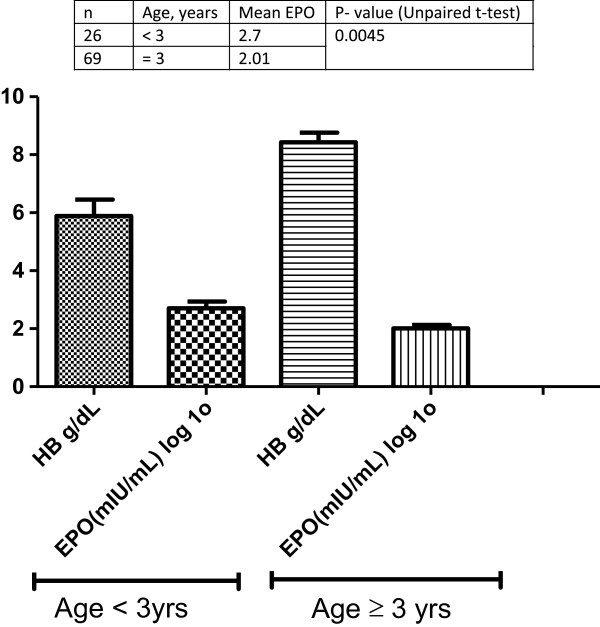
**The plasma levels of erythropoietin (log transformed) and haemoglobin levels in paediatric malaria cases for the study cohort categorised by age.**

To investigate the association among levels of erythropoietin, haemoglobin levels and age, venous blood samples were obtained from study participants and assayed for levels of eryrythropoietin. *P. falciparum* parasitaemia and haemozoin load in monocytes. There was an inverse correlation between age and Haemoglobin levels but this was not statistically significant. A scattergram revealed a similar trend using a scatterplot with multiple linear regressions with age and haemoglobin as independent variables (Figure 1). To investigate the age-related pattern of Erythropoietin production in paediatric malaria, graphpad prism 5 was used to draw a categorised bar chart of age for complicated and umcomplicated malaria with erythropoietin and haemoglobin as dependent variables. An upaired student t test revealed a significant difference in the mean production of erythropoietin in the different age groups (P = 0.005) with children aged < 3 years producing more erythropoietin compared to children aged greater than or equal to 3 years with relatively comparable levels of haemoglobin (Figure 2).

### Role of monocyte acquired haemozoin

**Figure 3:**
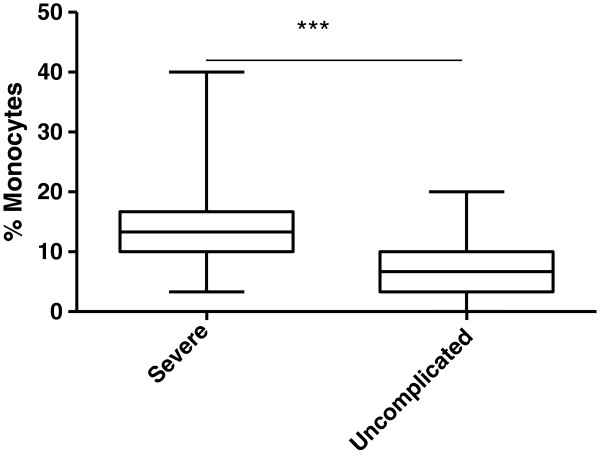
**Percentage of haemozoin containing monocytes in blood films of paediatric malaria patients.**

**Figure 4:**
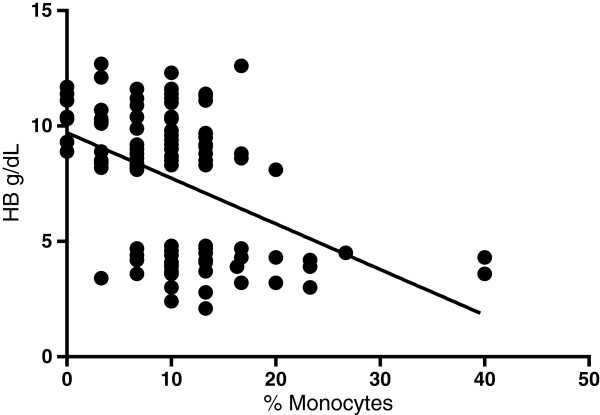
**Haemoglobin versus haemozoin containing monocytes.** In order to determine if haemozoin levels had any significant effect on haemoglobin levels, Pearsson correlation coefficient was determined. There was significant negative correlation between haemoglobin levels and percent monocytes (P < 0.0001) with r = −0.46.

To investigate the possible relationship between levels of haemozoin containing monocytes and severity of malaria cases, a boxplot was generated for levels of haemozoin containing monocytes in severe and uncomplicated malaria (Figure 3) This revealed a significant difference in percentage haemozoin containing monocytes in severe and uncomplicated malaria (P < 0.0001) with relatively higher levels of haemozoin containing monocytes in severe malaria anaemia (Figure 3). Also Hz + monocytes was negatively correlated with Hb levels (p < 0.0001) (Figure 4).

## Discussion

The study confirms the finding that younger children produce higher levels of erythropoietin compared to older children with a similar degree of anaemia (Hb levels) [12]. This finding is significant because it could partly explain the finding that severe malarial anaemia is more common in younger children whose bone marrow needs to be stimulated by high levels of EPO to replenish the loss of erythrocytes by producing new erythrocytes rapidly. Age and haemoglobin levels are reportedly independent variables with respect to erythropoietin responses. Apparently, erythropoietin response declines during development at a similar degree of anaemia. Age purportedly, has a direct effect on the background level of production of erythropoietin. With the corresponding decreased levels of erythropoietin in older children with the same degree of severe malarial anaemia, conceivably, the bone marrows of the younger children with acute malaria may be less sensitive to erythropoietin. Confirmation of this theory will require longitudinal studies including examination of bone biopsies from children with SMA, which is beyond the scope of the present preliminary, cross-sectional study.

Previous studies have established the ability of erythropoietin to cross the blood/brain barrier and to provide protection against experimental injury as well as act as a tissue protective cytokine [13, 14]. It is thus plausible to suggest that the high levels of erythropoietin in younger children with anaemia as compared to older children with relatively the same degree of anaemia may, presumably, protect them against tissue damage. It may also contribute to the low frequency of cerebral malaria in relatively younger children [13].

In the present study percentage haemozoin containing monocytes were significantly elevated in severe malaria anaemia compared to uncomplicated malaria. This finding corroborates earlier findings which implicated haemozoin in malaria pathogenesis through the suppression of erythropoiesis directly or in synergy with proinflammatory cytokines [9]. The elevated levels of haemozoin in severe malaria anaemia could therefore be accounted for by the increased pathogenesis therein. Besides the acquisition of haemozoin by human monocytes purportedly plays a crucial role in triggering severe malaria [10]. This study also showed a significant positive correlation between levels of erythropoietin and haemozoin containing monocytes. This corroborates earlier findings that demonstrated that haemozoin directly has an effect in the induction of apoptosis in nascent erythroid cells in malaria anaemia [15]. The inadequate erythropoietic response in the presence of high levels of erythropoietin, presumably results from the release of cytokines, chemokines and lipo-peroxides upon the exposure to haemozoin and these synergistically could lead to the concomitant inhibition of erythropoiesis [16, 17]. Taken together our findings are consistent with extant literature and support critical key findings in malaria pathogenesis that implicate haemozoin in the direct and indirect induction of dyserythropoiesis.

## Conclusion

In conclusion this study has confirmed earlier findings that erythropoietin production is age-related with younger children having the ability to produce more erythropoietin during acute malaria than older children. This study also suggests the involvement of haemozoin a parasite product in the inhibition of erythropoiesis as the seeming robust production of erythropoietin did not translate into higher Hb levels.
